# Evaluating the Conservation Status of a North-Western Iberian Earthworm (*Compostelandrilus cyaneus*) with Insight into Its Genetic Diversity and Ecological Preferences

**DOI:** 10.3390/genes13020337

**Published:** 2022-02-11

**Authors:** Daniel F. Marchán, Jorge Domínguez

**Affiliations:** 1 Centre d’Écologie Fonctionnelle et Évolutive (CEFE), Université Montpellier, 34000 Montpellier, France; 2 Grupo de Ecoloxía Animal (GEA), Universidade de Vigo, E-36310 Vigo, Spain; jdguez@uvigo.es

**Keywords:** biodiversity conservation, soil fauna, earthworms, genetic diversity, ecological niche modeling

## Abstract

In spite of the high conservation value of soil fauna, the evaluation of their conservation status has usually been neglected. This is more evident for earthworms, one of the most important ecosystem service providers in temperate habitats but rarely the subject of conservation research. These studies have not been developed in Western Europe, which comprises high diversity and several early-branching, relic genera. One potentially menaced representative of this fauna is *Compostelandrilus cyaneus*; this risk can be assessed by implementing potential distribution modeling and genetic diversity monitoring to their known populations. Genetic barcoding was performed in representatives of four populations (three of them newly sampled) in order to estimate genetic diversity and population genetics parameters. Ensemble species distribution models were built by combining several algorithms and using the five more relevant bioclimatic and soil variables as predictors. A large amount of genetic diversity was found in a small area of less than 20 km^2^, with populations located in less managed, better-preserved habitats showing higher genetic variability than populations isolated from natural habitats and surrounded by anthropic habitats. Potential distribution appears to be strongly restricted at a regional scale, and suitable habitats within the extent of occurrence appear fragmented and relatively limited. In addition, the main variables determining the ecological niche of *C. cyaneus* suggests a vulnerability to climate change and increasing soil compaction. Based on this knowledge, this species was assessed as Critically Endangered following the IUCN Red List of Threatened Species criteria, and some potential conservation actions are suggested.

## 1. Introduction

The conservation value of soil fauna has historically been disregarded, despite the ecological importance and high diversity of these animals [1,2]. It has been suggested that failure to preserve soil biodiversity could have drastic economic consequences [3] as well as affect ecosystem functioning and diversity [4].

Earthworms (Crassiclitellata, Annelida) constitute a special case within soil fauna in regard to their conservation value. The instrumental value of earthworms encompasses both direct and indirect economic benefits. The indirect economic value of earthworms as one of the most important ecosystem service providers in temperate habitats is widely known [5]: their activity modifies the physico-chemical properties of soil [6] and promotes microbial, animal, and vegetal diversity [7]. This may become even more relevant under global climate change and in the face of global food security risks [8,9]. Their direct economic benefits have less weight but are still important. Although direct consumption of earthworms is limited to some indigenous populations, “earthworm flour” is increasingly used as a protein source in human and animal nutrition [10]. Earthworms are also used as fish bait and in vermicomposting, both of which are economically important [11].

Even though comprehensive, large-scale works have been published on global patterns of earthworm diversity distribution [12], research on their conservation status is strikingly scarce relative to studies on other soil invertebrates (e.g., termites [13], Collembola [14], mites [15], myriapods [16]). Previous research of this type has mainly been carried out in the Balkans, New Zealand, and Australia. Stojanovic et al. [17,18,19] evaluated the rich endemic Lumbricidae fauna of the Balkans Peninsula, aiming to make up for the absence of red lists of earthworms in southeastern European countries. It was done at different scales: species level [17], genus level [18], and country-wide [19]. Going one step beyond scale-wise, Buckley et al. [20] published a systematized evaluation of the conservation status of New Zealand earthworms, establishing a template for further research in other countries and biogeographic regions. Due to its special status as a flagship species, the Giant Gippsland earthworm *Megascolides australis* has been the subject of particularly detailed conservation research [21,22], leading to a fully-fledged National Recovery Plan [23]. The basic biology, habitat preferences, and population structure of this species are fairly well known [24]. Preliminary genetic diversity assessment has also been conducted [25], although not publicly available.

The native earthworm fauna of Western Europe (belonging to the families Lumbricidae and Hormogastridae) is of great interest from evolutionary and ecological perspectives. The Iberian Peninsula (and Balearic Islands), southern France, Corsica, and Sardinia possess high diversity and endemicity and can be considered earthworm biodiversity hotspots. In addition, these terranes, which constituted a continuous landmass from the Cretaceous to the Oligocene-Miocene [26], have been suggested to be the center of origin of both sister families due to the presence of several early-branching genera [27,28]. Two of these genera, *Galiciandrilus* Domínguez, Aira, Porto, Díaz Cosín & Pérez-Losada 2017 and *Compostelandrilus* Domínguez, Aira, Porto, Díaz Cosín & Pérez-Losada 2017, are isolated in the NW Iberian Peninsula but appear to be closely related to *Kritodrilus* Bouché 1972 and *Vindoboscolex* Marchán 2021 in a surprisingly disjunct distribution [29]. This suggests that these taxa are relics of a formerly widely distributed lineage whose range may have contracted due to climate changes or replacement by other earthworm genera. The distribution of species of *Galiciandrilus* and *Compostelandrilus* appears to be extremely patchy and associated with habitats such as cork oak and holm oak forests, which are not widespread in the NW Iberian Peninsula. To add to the intrinsic conservation value of these species, each has highly divergent morphological characters, stressing the importance of each of them as reservoirs of the past morphological radiation of the aforementioned lineages.

*C. cyaneus* (Briones and Diaz Cosin, 1993) is the easternmost species of this complex, known from a single location in León (104 km from its closest relative). The surrounding area has been greatly modified by different types of agricultural land use, and the original habitats of this species have been reduced to hills and slopes unsuitable for crops. The species is, therefore, a priority target for conservation evaluation.

Potential distribution modeling/niche characterization [30,31,32] and genetic diversity monitoring [33] have been established as important tools for evaluating the conservation status of vertebrates. Ecological niche modeling has been used successfully on earthworms; in spite of their peculiarities (difficulty of sampling, patchy distributions), MaxEnt [34,35,36,37], Random Forests [38], and other algorithms have achieved high predictive power when using large-scale variables to predict the earthworms’ potential distribution. Implementing these approaches to earthworm biodiversity conservation would facilitate the necessary research on this key component of soil fauna.

The aims of this study are (i) to characterize the distribution and niche of *C. cyaneus*, (ii) to investigate the genetic diversity of known populations of this species, and (iii) to use this information for evaluating the conservation status of the species, establishing a template for other endemic earthworm species.

## 2. Materials and Methods

### 2.1. Specimens and Sampling

Specimens of *C. cyaneus* were collected in a sampling survey carried out in León, northwestern Spain, in November 2020. León is geologically heterogeneous, with three differentiated units: the Cantabrian mountain range to the north, the Bierzo depression and Galaico-Leonés mountain range to the west, and a Tertiary-Quaternary sedimentary basin in the rest of the province—the Meseta (plateau) [39]. In the Meseta (the main unit), the average elevation is between 700 and 1000 m, and the relief is near-horizontal, with elevated, softly undulated plains interrupted by fluvial valleys [40]. The general climate is Continental Mediterranean, with high thermal amplitude, long winters and irregular precipitations [41].

Thirty-one points were selected along two West–East transects and one North–South transect (connecting the former) in the area between the known populations of *Compostelandrilus*. Sampling points were spaced as regularly as possible (between 3 and 8 km), and natural-to-seminatural habitats were preferred (Table 1).

Habitat type and soil characteristics of the locations where *C. cyaneus* was found are as follows:

The north population (N) was sampled in a land mosaic of pastures with sclerophyllous vegetation and arable land in the close vicinity of the town of Villarmún. Soil characteristics: loam soil, coarse sand: 0%, fine sand: 47.63%, coarse silt: 8.014%, fine silt: 21.41%, clay: 22.94%, pHH (pH in H_2_O): 8.02, organic matter content: 2.0944%, nitrogen content: 0.1938%, C/N: 10.81.

The west population (W) was sampled within bushy pastures in an isolated hillock surrounded by non-irrigated arable land. Soil characteristics: clayey-sandy-loam soil, coarse sand: 0%, fine sand: 56.83%, coarse silt: 5.22%, fine silt: 9.15%, clay: 28.80%, pHH: 8.24, organic matter content: 1.45%, nitrogen content: 0.13%, C/N: 10.94.

The east population (E) was sampled within relatively unmodified sloping pastures with sclerophyllous vegetation. Soil characteristics: silty-loam soil, coarse sand: 11.28%, fine sand: 33.89%, coarse silt: 7.32%, fine silt: 43.82%, clay: 3.69%, pHH: 8.36, organic matter content: 1.59%, nitrogen content: 0.14%, C/N: 10.91.

The south population (S) was sampled within a large, continuous, sloping patch of pastures with sclerophyllous trees. Soil characteristics: fine sandy loam soil, coarse sand: 18.35%, fine sand: 35.64%, coarse silt: 5.41%, fine silt: 27.94%, clay: 12.66%, pHH: 8.26, organic matter content: 2.38%, nitrogen content: 0.25%, C/N: 9.58.

Earthworms were collected by digging and hand-sorting and were then rinsed with water and fixed in 96% ethanol. Sampling was qualitative, and a standardized sampling effort of one hour of digging by four researchers was applied to every location. The sampling and handling of specimens followed ethical considerations and conformed to Directive 2010/63/EU. The species were identified from the external and internal morphological characters analyzed by Domínguez et al. [42].

### 2.2. DNA Isolation and Sequencing

After morphological identification, up to 10 (preferentially mature) specimens of each population were chosen for further analysis. Total genomic DNA was extracted from ventral integument samples of approximately 5 mm × 5 mm using the DNeasy Blood & Tissue Kit (Qiagen). Cytochrome oxidase C subunit 1 (*COI*) was amplified by polymerase chain reaction (PCR), with the primers and conditions described by Pérez-Losada et al. [43]. PCR products were purified and sequenced by the C.A.C.T.I Genomics service (University of Vigo, Vigo, Spain).

DNA sequences obtained in this study are available in Genbank, and the accession numbers are shown in Table 1.

### 2.3. Phylogenetic Analyses and Population Genetics

Sequences from the other species of the genus *Compostelandrilus* (*menciae* and *bercianus*) generated in Domínguez et al. [42] were retrieved from Genbank and used as a reference for the phylogenetic analysis.

Sequences were aligned using MAFFT v.7 [44], with default settings. After trimming, the obtained sequences had a length of 606 base pairs. The best-fit evolutionary model was selected using jModelTest v. 2.1.3 [45] by applying the Akaike information criterion (AIC) [46], Bayesian information criterion (BIC) [47], and the Decision Theory method (DT). GTR + I + G was selected as the best-fit evolutionary model.

Maximum Likelihood phylogenetic inference was performed using RAxML-NG [48] in the CIPRES Science Gateway V. 3.3 platform, from 10 random starting trees and 1000 rapid bootstrap replicates. A suitable starting tree for the time-calibrated phylogenetic inference was generated by using the chronopl function in the R package *ape* v5.2. to convert the Maximum Likelihood tree into an ultrametric tree by non-parametric rate smoothing (NPRS). A relative calibration (root age = 1) was implemented: this approach provides an approximate visualization of the relative age of the clades without relying on external calibrations or assumptions of vicariance.

A uniform distribution with an initial value = 0.002 (ranging from 0.00005 to 0.02) was specified through the ucld.mean parameter, and a uniform distribution with an initial value = 0.10 (ranging from 0 to 10) was specified for the ucld.stdev parameter. A relative calibration of 1 (mean = 1, standard deviation = 0.05) was implemented as a normal prior for the root of the tree. Fifty million generations were specified for the Monte-Carlo Markov chain, and sampling was conducted every 5000th generation. The log file was visualized in Log Tracer v. 1.7 [49] to check for convergence and effective sampling sizes over 100. The final tree was generated using TreeAnnotator v.1.10.4. [50] with a burn-in of 2000 trees.

Haplotype networks were obtained using PopART 1.7 [51] for graphical representation.

Genetic diversity and population genetics parameters were determined using DnaSP 6 [52]: haplotypic diversity (H), nucleotide diversity (π), Tajima’s D, Fu’s Fs, Fu & Li’s D* and Fu & Li’s F*.

### 2.4. Species Distribution Modelling

Twenty large-scale variables were chosen as putative predictor variables: the suite of bioclimatic variables BIO1-BIO19 was downloaded from Worldclim (available online: http://www.worldclim.org/, accessed on 1 December 2020), and Corine Land Cover 2018 (100 m × 100 m resolution) (available online: https://land.copernicus.eu/pan-european/corine-land-cover/clc2018?tab=download (accessed on 3 January 2022)) was chosen to represent land use and vegetation type.

The following six soil variables from SoilGrids [53], corresponding to a depth of 10 cm, were downloaded: soil bulk density (available online: https://doi.org/10.5281/zenodo.2525665 (accessed on 3 January 2022)), clay content (available online: https://doi.org/10.5281/zenodo.2525663 (accessed on 3 January 2022)), sand content (available online: https://doi.org/10.5281/zenodo.2525662 (accessed on 3 January 2022)), organic carbon content (available online: https://doi.org/10.5281/zenodo.2525553 (accessed on 3 January 2022)), soil water content (available online: https://doi.org/10.5281/zenodo.2784001 (accessed on 3 January 2022)), and soil pH (available online: https://doi.org/10.5281/zenodo.2525664 (accessed on 3 January 2022)).

All layers were aggregated to the smallest resolution possible (100 m × 100 m).

### 2.5. Variable Selection

In order to select the most suitable predictor variables, a Boosted Regression Tree analysis was performed following a modified version of the script by Irving et al. [54] in R. After identifying the variables of the highest relative importance, correlation analysis was performed, and variables with correlation values above 0.7 were discarded. A total of five variables were chosen for the final dataset:

− BIO 4: Temperature Seasonality (TEMPSEA)
− BIO 15: Precipitation Seasonality (PRSEA)
− BIO 8: Mean Temperature of Wettest Quarter (MTWQ)
− Soil water content (SOILW)
− Soil bulk density (SOILBD)

Ecological niche models were obtained using the R package ‘SSDM’ [55] with standard parameters. Ensemble species distribution models (ESDMs) were built by combining the algorithms (‘MAXENT’, ‘GLM’, ‘CTA’, ‘MARS’, ‘SVM’, ‘GBM’, ‘GAM’ and ‘ANN’), producing kappa values greater than 0.5, with 5 repetitions for each algorithm. Both a presence–absence analysis and a presence-only analysis with randomly generated pseudoabsences were performed and were compared according to the evaluation parameters provided by package ‘SSDM’ by default (area under the curve—AUC, omission rate, sensitivity, specificity, proportion of correctly predicted occurrences, and Kappa).

Extent of occurrence (EOO) and area of occupancy (AOO) were calculated in QGIS 3.16.3. The area of suitable habitat was determined according to the dominant land use. The latter was obtained from SIGPAC (Agricultural Plot Geographic Information System) for 2012 and 2020 for calculation of potential habitat loss.

## 3. Results

### 3.1. Sampled Populations

All of the individuals studied possessed internal and external morphological characters consistent with the original description [56] and with that of Domínguez et al. [42].

For the north population (N), a total of 23 individuals were sampled: 1 mature, 16 semi-mature (showing developed tubercula pubertatis and hints of clitellum), and 6 immature. For the west population (W), 8 individuals were sampled: 5 semi-mature and 3 immature. For the east population (E), 10 individuals were sampled: 4 semi-mature and 6 immature. For the south population (S), 10 individuals were sampled: 1 mature, 5 semi-mature and 4 immature.

### 3.2. Phylogenetic Analyses and Population Genetics

**Figure 1:**
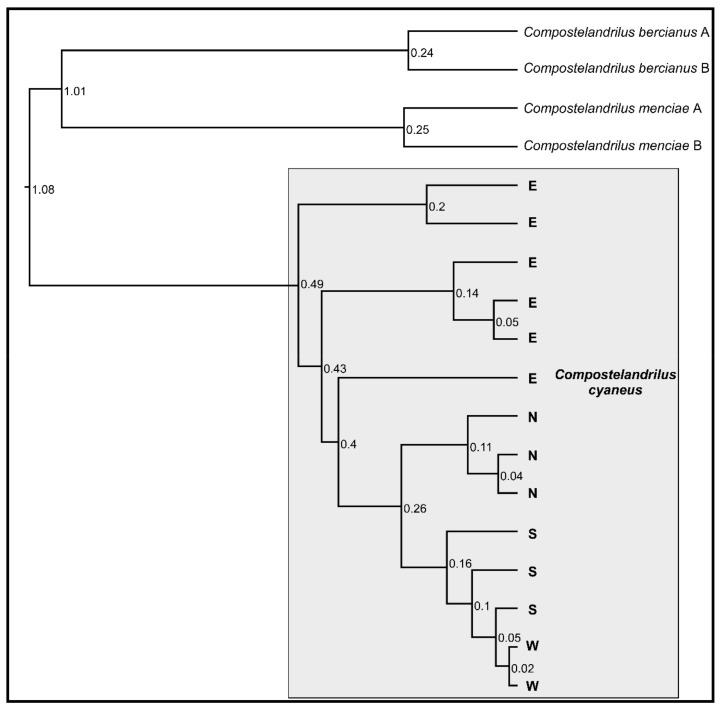
Ultrametric tree based on the COI sequences of the different populations of *C. cyaneus* and their closest relatives. All nodes showed posterior probability and support values over 90. Relative ages are shown at each node. N—North population, S—South population, W—West population, E—East population.

The four populations of *C. cyaneus* were recovered as a sister clade to *C. bercianus* and *C. menciae* by the phylogenetic analysis (Figure 1). The four populations were not recovered as monophyletic: the N, S, and W populations were nested within paraphyletic population E, divided into three deep lineages. In addition, population W was nested within paraphyletic population S (Figure 1). The estimated relative age for the different lineages within population E was older than the split between the known populations of *C. bercianus* and *C. menciae*, while the split between populations N and S showed a similar estimated age to the latter. The split of population W within population S appeared, proportionately, very recent.

Uncorrected average pairwise genetic (UAPG) distance based on the COI sequences between the four populations ranged between 1.08% and 5.78% (Table 2). The lowest value corresponded to the divergence between the W and S populations, while the highest values corresponded to the divergence between the E and the S–W populations. Intrapopulation UAPG divergence ranged between 0.04% and 0.96%.

**Figure 2:**
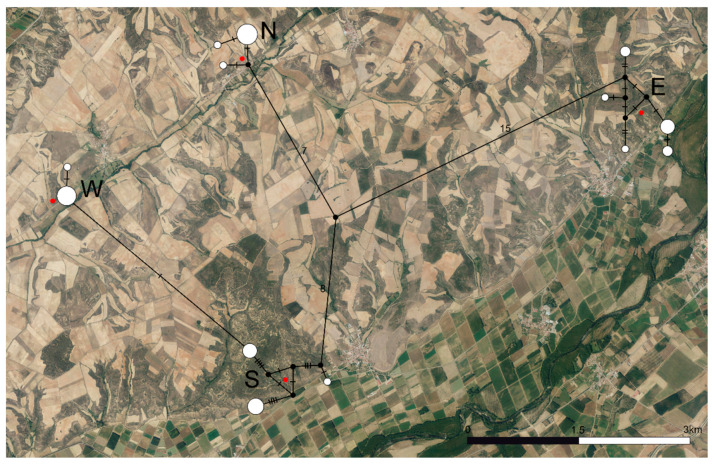
Haplotype network based on the COI barcode region for the four populations of *C. cyaneus* under study. Black dots represent inferred missing haplotypes. Red dots indicate sampling locations. Numbers and dashes represent mutational steps between each haplotype sampled. The size of the circles is proportional to the number of individuals sharing each haplotype. N—North population, S—South population, W—West population, E—East population.

The haplotype network (Figure 2) displayed complex genetic structures within populations S and E, while they were significantly simpler for populations N and W. Populations E, N, and S were separated by 22, 23, and 15 mutational steps, respectively, while populations S and W were separated by a single mutational step.

Haplotypic diversity (H) (Table 3) was high for populations S and E (0.644–0.822) and low for populations W and N (0.250–0.378). Nucleotide diversity (π) was low for all populations (0.00099–0.00957). The H-π relationships for populations S and E (high H and low π) corresponded to high demographic expansion from small effective populations. For populations W and N, H–π relationships (low–low) matched the expectation for recent bottlenecks or founder effects.

None of the estimated demographic parameters (Tajima’s D, Fu’s Fs, Fu & Li’s D*, Fu & Li’s F*) were statistically significant.

### 3.3. Species Distribution Modeling

Both ecological niche models obtained (presence–absence and presence-only) displayed high predictive power, with high AUC and kappa values (0.985–0.99 and 0.90–0.92, respectively), high sensitivity and specificity (1–1 and 0.97–0.99, respectively), and low omission rates (0.00) (Table S1).

**Figure 3:**
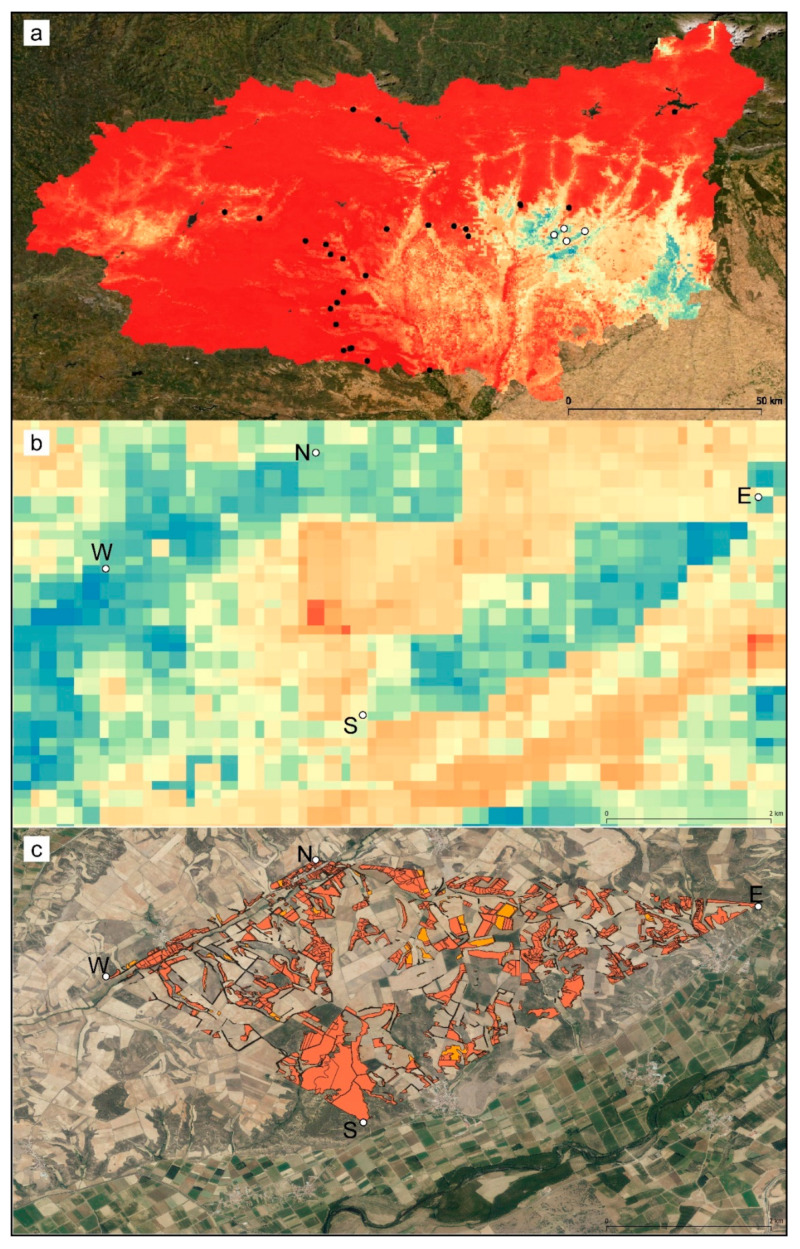
Geographical representation of the predicted suitability values estimated by the presence–absence ensemble distribution model for *C. cyaneus*; (**a**) province-wide and (**b**) detail of the extent of occurrence. The lowest values are shown in red, and the highest values are shown in blue. White dots indicate presence locations, and black dots indicate absence locations. (**c**) Dominant land use plots (according to SIGPAC) corresponding to the preferred habitat of *C. cyaneus*; Orange: 2012; red: 2020. N—North population, S—South population, W—West population, E—East population.

The geographical representation of the predicted suitability values of the presence–absence model is shown in Figure 3. The predicted highly suitable areas were narrower than in the presence-only model (Figure S1) and restricted to a narrow southeastern band in the province of León.

The relative contributions of the predictor variables to each model are shown in Table S1. Temperature Seasonality, Precipitation Seasonality, and Soil Bulk Density were the three most influential variables for both models.

The extent of occurrence (EOO) (the polygon delimiting the known populations) covered an area of 17.48 km^2^. The area of suitable habitat, according to the SIGPAC dominant land-use types (pastures, pastures with bushes, pastures with trees; Figure 3c), was 4.2 km^2^ in 2012 and 4.5 km^2^ in 2020, i.e., 24% and 26% of the EOO. These areas can be considered estimates of the area of occupancy (AOO). There was no estimated loss of suitable habitat in the 2012–2020 period.

## 4. Discussion

### 4.1. Genetic Diversity

The information obtained about the genetic diversity of the known populations of *C. cyaneus* can be used to infer the long-term viability and connectivity of the populations and their relationship with the environment.

For such a small area (less than 20 km^2^), known populations of *C. cyaneus* harbor a large amount of genetic diversity. Populations E and S, located in less managed, better-preserved habitats, showed the highest amount of genetic variability; on the other hand, populations W and N, which were isolated from natural habitats and surrounded by anthropic habitats (non-irrigated arable land), showed significantly lower genetic diversity. Together with population genetic parameters, this suggests that the latter populations colonized their current habitats from the southeastern populations and became isolated by land-use changes and a reduction in suitable habitat. The reduction in genetic diversity may be explained by low connectivity with other populations and lower habitat suitability [57,58,59,60]. If these factors lead to inbreeding depression, the populations may be at a higher risk of extinction [61].

Genetic diversity appeared uncorrelated with the five environmental variables selected for ecological niche modeling. It could have been expected that, at least for the three most influential variables (seasonality of temperature and precipitation and soil bulk density), genetic diversity would be higher for certain preferred values of these variables; yet, such an effect was not observed. It is possible that the geographical distance between the populations is too small for them to display significant environmental heterogeneity. Another possibility is that a single molecular marker is not enough to capture the effect of such an environmental pressure on genetic diversity; landscape genomics analyses (such as in [62]) could help to test this hypothesis.

### 4.2. Ecological Niche and Distribution

The potential distribution of *C. cyaneus* appears to be strongly restricted at a regional scale, corresponding to roughly 10% of the area of the province of León.

Even without taking land use into account (as Corine Land Cover was excluded in the selection step), suitable habitat within the EOO appears fragmented and relatively limited. Considering the main variables determining the ecological niche of *C. cyaneus* (seasonality of temperature and precipitation and soil bulk density), climate change and increasing soil compaction may have historically modified the area of suitable habitat for this species and may further modify it in the future. These are two of the main threats to soils and to agriculture sustainability [63,64].

Suitable habitat, as inferred from the preferred types of land use (pastures with or without bushes or trees), also shows a high degree of fragmentation, with large gaps of unfavorable habitat between patches. While preferred habitats do not appear to have decreased in the last 8 years, they are evidently scarce in the area, as arable land and reforested woodland appear to be the dominant types of land use.

Due to the characteristics of their habitat, ecological preferences and the fine-scale distribution of soil fauna are generally poorly known when compared with more conspicuous animals; in fact, soils have been described as the “third biotic frontier” (after oceanic abysses and tropical forest canopies) [65]. Ecological niche modeling provides a useful tool for opening such a black box. The identification of the most relevant variables for the potential distribution of soil species can serve as a starting point for experimental work on the interaction between environmental variables and life-history traits. In addition, delimiting potentially highly suitable habitats should facilitate the rigorous sampling of these animals at a large scale by discarding a priori wide unsuitable areas and focusing the sampling effort on those most likely to contain the target species [31]. This is important in order to make quantitative sampling across the range of an earthworm species viable. Although qualitative sampling with standardized effort is suitable for the evaluation of earthworm populations, quantitative sampling should be the preferred choice: it unlocks information such as abundance and population density, which can be compared between populations and correlated to habitat characteristics and large-scale environmental variables [66].

### 4.3. Conservation Status Evaluation for C. cyaneus

The following assessment roughly follows the structure and format of the IUCN Red List of Threatened Species.

**Taxonomy**

**Kingdom Animalia, Phylum Annelida, Order Megadrili, Family Lumbricidae**

Taxon Name: *Compostelandrilus cyaneus* (Briones and Diaz Cosin, 1993)

Taxonomic Source(s):

Domínguez, J., Aira, M., Porto, P. G., Díaz Cosín, D. J., & Pérez-Losada, M. (2018). Multigene phylogeny reveals two new isolated and relic earthworm genera (Oligochaeta: Lumbricidae). *Zoological Journal of the Linnean Society*, *182*(2), 258-274.

Jiménez, S., Marchán, D.F., Novo, M., Trigo, D., Domínguez, J., Díaz Cosín, D.J. (2021). Sorry atlanticus, you are not my type. Molecular assessment splits *Zophoscolex* (Lumbricidae, Crassiclitellata) into French and Iberian genera. *Zoological Journal of the Linnean Society.*

**Assessment Information**

Red List Category & Criteria: Critically Endangered B1ab(iii)+2ab(iii) ver 3.1

Justification:

This species is assessed as Critically Endangered owing to the restricted extent of occurrence (EOO), estimated area of occupancy (AOO), the expected decline in quality of habitat, and occurrence in only four locations.

**Geographic Range**

Range Description:

This species has only been found in four locations in the province of León (Castilla y León, Spain), in the vicinities of the localities of Villarmún, Valle de Mansilla, San Miguel de Escalada and Palazuelo de Eslonza.

The extent of occurrence (EOO) for this species is 17.48 km², and the estimated area of occupancy (AOO) is 4.5 km^2^.

Country occurrence:

Native: Spain

**Distribution Map**

See maps in Figure 2 and Figure 3.

**Population**

Four populations of this species have been discovered.

Current population trend: unknown. Population genetics suggest that two of the known populations display low genetic diversity and may have suffered recent bottlenecks.

**Habitat and Ecology**

The species has been found in natural pastures with sclerophyllous bushes and/or trees (*Quercus ilex rotundifolia*), usually restricted to isolated hillocks and slopes when the habitat is surrounded by arable land. It has not been found in reforested woods composed of pines or other non-native, commercial species. Other earthworm species are rarely found within the same patches as *C. cyaneus*, the most frequent being *Aporrectodea trapezoides* (Dugès, 1828) and *Aporrectodea rosea* (Savigny, 1826).

**Figure 4:**
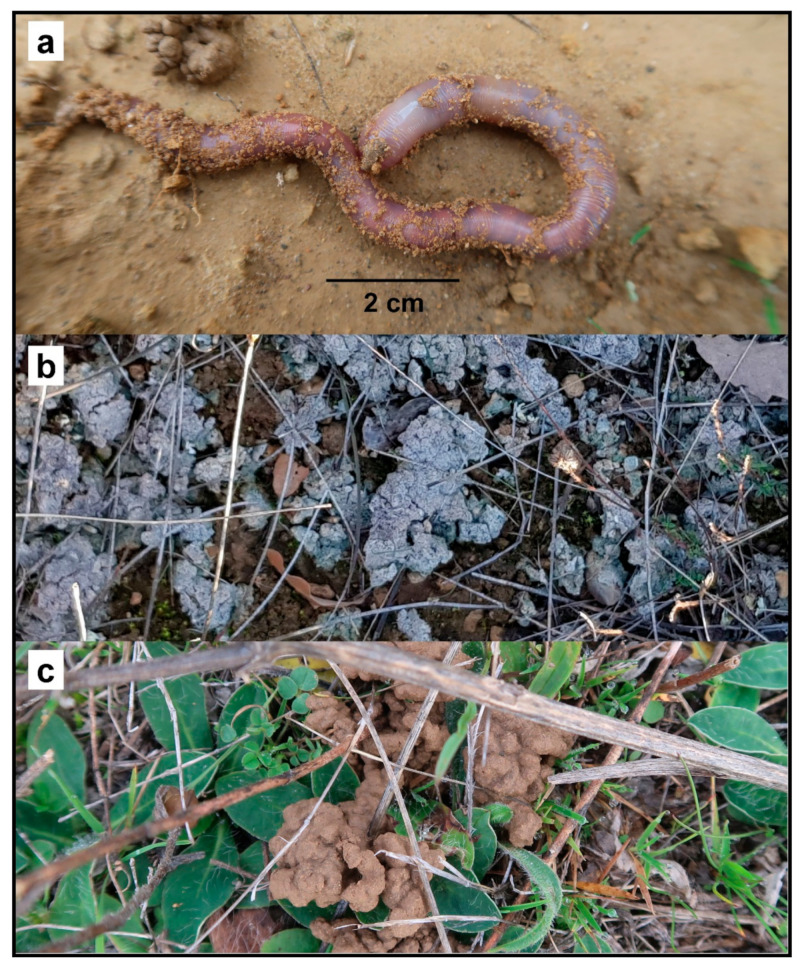
(**a**) Live specimen of *C. cyaneus*. (**b**) Surface casts of *C. cyaneus* colonized by lichens. (**c**) Surface casts of *C. cyaneus*.

*C. cyaneus* is a large endogeic earthworm (Figure 4a). This ecological category is usually associated with poor active dispersal abilities (as these earthworms move slowly and rarely crawl over the surface) and k-like life-history strategies (long time to reach maturity, low number of offspring, high longevity). *C cyaneus* deposits a large volume of casts on the soil surface: this is very likely to modify the resistance of soils to erosion by surface runoff. The casts appear to be later colonized by unidentified lichens (Figure 4b).

Systems: terrestrial

**Use and trade**

The species is not utilized in any known form.

**Threats**

The small area inhabited by this species has been greatly modified by agricultural land use, and populations are usually immersed in mosaics of natural and anthropogenic habitats. An increase in the area dedicated to crops or reforested woods would reduce the suitable habitat for these earthworms, further reduce the connectivity between populations, and increase putative competition with cosmopolitan earthworms.

**Conservation Actions**

No conservation actions are currently in place for this species as the populations are not included within any protected areas. Further sampling within and around the EOO should be conducted to confirm the extent of the area and to provide further information about the preferred habitats of the species. A citizen science program (possibly including local farmers) could be developed, owing to the easy recognition of the surface casts of this species (Figure 4c). This would enable the monitoring of populations. A plan limiting the establishment of non-native tree plantations and arable land within the habitats of the known populations and encouraging the creation of corridors of pastures with sclerophyllous bushes and trees between the known populations should be considered priority actions by the relevant authorities.

### 4.4. Implications for Earthworm Conservation

Even though different earthworm species show significant differences in ecological preferences [67] and their response to habitat alteration and other human-mediated impacts [68], some implications about potential threats and possible conservation actions could be extrapolated to related or ecologically similar earthworm taxa.

The preservation of patches of original, unmodified habitat appears to be key to narrowly distributed, endogeic earthworms such as *C. cyaneus* as they act as genetic diversity reservoirs from which the recolonization of surrounding habitats could be eventually achieved. To avoid isolation between those patches and recently established populations that are in genetic drift, with reduced fitness and eventual extinction, connectivity between populations should be encouraged. For that, corridors of native vegetation should be preserved or restored. The importance of hedgerows and grass field margins for invertebrate diversity [69] and soil functioning [70] is well known, but their putative function as genetic flow enablers could give them additional value for soil fauna conservation. The lower bulk density in relation to other land uses [70] should make them highly suitable habitats for earthworm species that share similar ecological preferences to *C. cyaneus.*

Even though native, endemic species can be occasionally found in unfavorable land uses such as arable lands, they are usually outcompeted by exotic or cosmopolitan earthworms in such highly-productive soils [71], hindering their recolonization by the former. However, in soils with lower availability of resources, native earthworms may prevent cosmopolitan earthworms from further expanding their range [71]. This dynamic further highlights the importance of land-use mosaics, including grass field margins and hedgerows.

## 5. Conclusions

Genetic diversity estimation based on COI molecular barcoding and ecological niche modeling based on large-scale and soil predictor variables has provided useful insight into the conservation status of a narrowly distributed, endemic earthworm from the highly diverse Iberian Peninsula. The more vulnerable populations were identified based on low genetic diversity and habitat isolation. In addition, threats for its populations were inferred from the most influential environmental variables. This approach appears promising in terms of facilitating research on earthworm conservation evaluation and compensating for the lack of attention to this key element of soil ecosystems in conservation biology.

## Figures and Tables

**Table 1:** Sampling locations of the four known populations of *Compostelandrilus cyaneus* and the corresponding Genbank accession numbers for the COI sequences obtained. Locations where *C. cyaneus* was absent are also listed.

Population	Location	Latitude	Longitude	Accessions
N—North	Villarmún, León, Spain	42.5768	−5.372	MZ614758-67
S—South	Valle de Mansilla, León, Spain	42.538	−5.3649	MZ614768-77
E—East	San Miguel de Escalada, León, Spain	42.5703	−5.3064	MZ614786-95
W—West	Palazuelo de Eslonza, León, Spain	42.5596	−5.4032	MZ614778-85
Absences	—	−5.9917	42.1605	
	—	−6.0466	42.1997	
	—	−6.0415	42.2014	
	—	−6.0664	42.194	
	—	−6.0903	42.2758	
	—	−6.1084	42.3257	
	—	−6.0873	42.3445	
	—	−6.0671	42.3783	
	—	−5.9968	42.4307	
	—	−5.7951	42.1316	
	—	−5.3551	42.6444	
	—	−5.51	42.6553	
	—	−5.5077	42.6524	
	—	−5.6802	42.5769	
	—	−5.6727	42.5544	
	—	−5.7181	42.5859	
	—	−5.7992	42.5892	
	—	−6.4417	42.6305	
	—	−6.3316	42.6109	
	—	−6.187	42.5397	
	—	−6.1226	42.5281	
	—	−6.1077	42.497	
	—	−6.0688	42.4837	
	—	−5.9306	42.5774	
	—	−6.0362	42.954	
	—	−5.9577	42.9222	
	—	−5.0223	42.9468	

**Table 2:** COI uncorrected average pairwise genetic (UAPG) distance between the four populations of *C. cyaneus* under study, expressed as percentage. The UAPG distances within populations are shown in bold.

	N	S	W	E
N	**0.1**			
S	4.03	**0.96**		
W	4.16	1.08	**0.04**	
E	4.22	5.65	5.78	**0.5**

**Table 3:** Genetic diversity and population genetics parameters obtained for each of the main populations under study. *P*-values were not significant (>0.10) for any of the comparisons. N: number of sequences. Nh: number of haplotypes. H: haplotypic diversity, π: nucleotide diversity, D: Tajima’s D, Fs: Fu’s Fs, D*: Fu & Li’s D*, F*: Fu & Li’s F*.

	N	Nh	H	π	D	Fs	D*	F*
N	10	3	0.378	0.00099	−1.5622	−0.459	−1.7844	−1.9338
S	10	3	0.644	0.00957	0.7877	5.482	0.137	0.33603
W	8	2	0.25	0.00041	−1.0548	−0.182	−1.1264	−1.2035
E	10	5	0.822	0.00499	−0.2191	0.34	0.21714	0.12306

## Data Availability

The datasets generated during and/or analyzed during the current study are available from the corresponding author on reasonable request. Molecular barcoding sequences are available in Genbank under accession numbers MZ614758-MZ614795.

## Supplementary Materials

The following supporting information can be downloaded at: https://www.mdpi.com/article/10.3390/genes13020337/s1, Table S1: Predictive model parameters and relative contributions of the predictor variables. AUC: area under the curve, prop.correct: proportion of correctly predicted occurrences, TEMPSEA: temperature seasonality, PRSEA: precipitation seasonality, MTWQ: mean temperature of wettest quarter, SOILW: soil water content, SOILBD: soil bulk density. Figure S1: Geographical representation of the predicted suitability values estimated by the presence-only ensemble distribution model for *C. cyaneus*. The lowest values are shown in red, and the highest values are shown in blue. White dots indicate presence locations, and black dots indicate absence locations.
